# Supplementary material for: A Propensity Score Matched Analysis of Superparamagnetic Iron Oxide versus Radioisotope Sentinel Node Biopsy in Breast Cancer Patients after Neoadjuvant Chemotherapy
Source: Cancers (Basel). 2022 Jan 28;14(3):676. doi: 10.3390/cancers14030676 (PMC8833727; doi:10.3390/cancers14030676)
Supplement: Supplementary file 1 [file cancers-14-00676-s001.zip › cancers-1539357-supplementary.pdf]

Supplementary Materials

A Propensity Score Matched Analysis of Superparamagnetic Iron Oxide versus Radioisotope Sentinel Node Biopsy in Breast Cancer Patients after Neoadjuvant Chemotherapy

Zuzanna Pelc [1,*], Magdalena Skórzewska [1], Maria Kurylcio [1], Tomasz Nowikiewicz [2], Radosław Mlak [3], Katarzyna Sędłak [1], Katarzyna Gęca [1], Karol Rawicz-Pruszyński [1], Wojciech Zegarski [2], Wojciech P. Polkowski [1] and Andrzej Kurylcio [1]

[1] Department of Surgical Oncology, Medical University of Lublin, Radziwiłłowska 13 St., 20-080 Lublin, Poland; magdalena.skorzewska@umlub.pl (M.S.); m.kurylcio@gmail.com (M.K.); sedlak.katarz@gmail.com (K.S.); kasiaa.geca@gmail.com (K.G.); karolrawiczpruszynski@uml.edu.pl (K.R.-P.); wojciechpolkowski@umlub.pl (W.P.P.); andrzej.kurylcio@umlub.pl (A.K.)

[2] Department of Clinical Breast Cancer and Reconstructive Surgery, Oncology Center, Prof. Lukaszczyk Memorial Hospital, Romanowskiej 2 St., 85-796 Bydgoszcz, Poland; tomasz.nowikiewicz@cm.umk.pl (T.N.); zegarskiw@cm.umk.pl (W.Z.)

[3] Department of Human Physiology, Medical University of Lublin, Radziwiłłowska 11 St., 20-080 Lublin, Poland; radoslaw.mlak@gmail.com

* Correspondence: zuzanna.torun@gmail.com; Tel.: +48-81-53-18-126

Table S1. Influence of selected demographic and clinical variables on the efficacy of LNs evaluation in the RI and SPIO groups.

Variable	RI (n = 62)		p^a lub OR [95%CI] p^b	SPIO (n = 62)		p^a lub OR [95%CI] p^b
	SLNs Evaluation Efficacy			SLNs Evaluation Efficacy		
	<3 evaluated LNs	>=3 evaluated LNs		<3 evaluated LNs	>=3 evaluated LNs	
**Age (lyears)** Median [interquartile range]	55.5 [49–62]	47.5 [43–60]	0.0618	52 [43–59]	55 [43.5–62.5]	0.6674
**Weight (kg)** Median [interquartile range]	71 [60–78]	69 [62–73]	0.5349	69 [56–85]	67 [60.5–77]	0.7680
**BMI** Median [interquartile range]	25.94 [23.19–28.76]	25.53 [23.46–29.69]	0.7781	25.90 [21.60–30.12]	25.97 [21.91–28.30]	0.9798
**BMI**						
Underweight or Healthy body weight	13 (48.1%)	14 (51.9%)	0.98 [0.36–2.68] 0.9736	6 (25%)	18 (75%)	1.25 [0.37–4.19] 0.7176
Overweight or Obese (classes 1–3)	17 (48.6%)	18 (51.4%)		8 (21.1%)	30 (78.9%)	

**Tumor highest diameter (mm)**	16 [13–31]	22 [13–30]	0.7969	25 [19–34]	22 [13–30]	0.3580
**ypT**<br>0<br>1 or 2	17 (47.2%)<br>13 (50%)	19 (52.8%)<br>13 (50%)	0.89 [0.33–2.45]<br>0.8290	8 (22.9%)<br>6 (22.2%)	27 (77.1%)<br>21 (77.8%)	1.04 [0.31–3.45]<br>0.9527
**ypN**<br>Negative<br>Positive	27 (47.4%)<br>3 (60%)	30 (52.6%)<br>2 (40%)	0.60 [0.09–3.87]<br>0.5910	13 (22.8%)<br>1 (20%)	44 (77.2%)<br>4 (80%)	1.18 [0.12–11.52]<br>0.8857
**Biological subtype of cancer**<br>A, B1, B2, HER2+<br>TN	21 (47.7%)<br>9 (50%)	23 (52.3%)<br>9 (50%)	0.91 [0.30–2.73]<br>0.8709	11 (25%)<br>3 (16.7%)	33 (75%)<br>15 (83.3%)	1.67 [0.40–6.86]<br>0.4792
**Biological subtype of cancer**<br>A, B1, B2, TN<br>HER2+	27 (49.1%)<br>3 (42.9%)	28 (50.9%)<br>4 (57.1%)	1.29 [0.26–6.29]<br>0.7564	13 (23.6%)<br>1 (14.3%)	42 (76.4%)<br>6 (85.7%)	1.86 [0.20–16.87]<br>0.5824
**Response to NAC***<br>No response<br>Response	9 (47.4%)<br>21 (48.8%)	10 (52.6%)<br>22 (51.2%)	0.94 [0.32–2.78]<br>0.9150	6 (23.1%)<br>8 (22.2%)	20 (76.9%)<br>28 (77.8%)	1.05 [0.31–3.50]<br>0.9367

RI—radioisotope, SPIO—superparamagnetic iron oxide, OR—odds ratio, pa—chi-square test result, pb—odds ratio test result, SLNs—sentinel lymph nodes, BMI—body mass index, ypT—post neoadjuvant therapy pathological tumor categorization, ypN—post neoadjuvant therapy pathological nodal categorization, A—luminal A, B1—luminal B human epithelial receptor-negative, B2—luminal B human epithelial receptor-positive, HER2+—human epithelial receptor-positive, TN – triple-negative, *—statistically significant result, ^—a trend into statistically significant result.
